# Differential Cold Tolerance on Immature Stages of Geographically Divergent *Ceratitis capitata* Populations

**DOI:** 10.3390/biology12111379

**Published:** 2023-10-27

**Authors:** Antonis G. Papadopoulos, Panagiota Koskinioti, Kostas D. Zarpas, Nikos T. Papadopoulos

**Affiliations:** Department of Agriculture, Crop Production and Rural Environment, School of Agricultural Sciences, University of Thessaly, 38446 Volos, Greece; antoniopap@uth.gr (A.G.P.); pakoskin@uth.gr (P.K.); kzarp@uth.gr (K.D.Z.)

**Keywords:** immatures, acute cold stress, subfreezing temperatures, biotypes, Diptera, Tephritidae, thermal biology

## Abstract

**Simple Summary:**

*Ceratitis capitata*, also known as the Mediterranean fruit fly, is a notorious pest of fruits and vegetables worldwide. The current expansion of its geographic distribution is limited by the cooler temperate areas of the Northern Hemisphere, which points to a more comprehensive investigation of its thermal biology considering its high invasion capacity. Cold tolerance in adults has been studied thoroughly but the impact of extremely low temperatures on the immature stages remains unexplored. We estimated the acute cold stress response of three geographically divergent populations (two from Greece and one from Croatia). Overall, the populations exhibited different responses to acute cold stress depending on the developmental stage. The northernmost population was the most cold-susceptible at the egg stage, whereas in the southernmost population it was at the larval and pupal stage. The geographically intermediate population was the most cold-tolerant regardless of the developmental stage. The egg stage was the most cold-tolerant, followed by pupae and larvae. Our findings broaden the existing knowledge on cold tolerance of *C. capitata* and can be used for the development of population and invasion dynamics models.

**Abstract:**

Cold tolerance of adult medflies has been extensively studied but the effect of subfreezing temperatures on the immature stages remains poorly investigated, especially as far as different populations are regarded. In this study, we estimated the acute cold stress response of three geographically divergent Mediterranean fruit fly populations originating from Greece (Crete, Volos) and Croatia (Dubrovnik) by exposing immature stages (eggs, larvae, pupae) to subfreezing temperatures. We first determined the LT_50_ for each immature stage following one hour of exposure to different temperatures. Then eggs, larvae and pupae of the different populations were exposed to their respective LT_50_ for one hour (LT_50_ = −11 °C, LT_50_ = −4.4 °C, LT_50_ = −5 °C for eggs, larvae and pupae, respectively). Our results demonstrate that populations responded differently depending on their developmental stage. The population of Dubrovnik was the most cold-susceptible at the egg stage, whereas in that of Crete it was at the larval and pupal stage. The population of Volos was the most cold-tolerant at all developmental stages. The egg stage was the most cold-tolerant, followed by pupae and finally the 3rd instar wandering larvae. This study contributes towards understanding the cold stress response of this serious pest and provides data for important parameters that determine its successful establishment to unfavorable environments with an emphasis on range expansion to the northern, more temperate regions of Europe.

## 1. Introduction

Temperature (extremes, averages and fluctuations) adversely affecting physiological and biochemical processes [1,2,3] is the main abiotic factor that determines the survival, development, growth, reproduction and population dynamics of poikilotherms [2,4,5,6,7]. Because of climate change, many tropical insect pests have expanded their geographic distribution limits by invading cooler, more temperate areas of the Northern Hemisphere [8]. Dispersal and successful establishment in novel habitats entail adaptation strategies including physiological, morphological and behavioral alterations which enable these organisms to (a) overcome a substantial number of obstacles they encounter to rapidly shifting environments [9,10,11,12,13,14], (b) resist unfavorable conditions [15,16,17,18] and ultimately (c) persist and thrive [19].

True fruit flies (Diptera: Tephritidae) include major invasive species such as the Mediterranean fruit fly (medfly) *Ceratitis capitata* (Wiedemann) (Diptera: Tephritidae) [20], a tremendously devastating, polyphagous and multivoltine invasive species of fresh fruits and vegetables worldwide [21,22,23,24,25,26]. Originating in Eastern Sub-Saharan Africa [8,26,27,28], medfly has managed to rapidly colonize the entire African continent [29], invade the Mediterranean Basin [8,30] and from there the Middle East [22], Latin America (first record in Brazil in 1905) [31] and western Australia [32,33]. In more recent times, it has expanded its northern distribution limits to southern France [34] and Northern Italy [35,36,37,38,39] where established populations have been recorded. Today, the existence of medfly has been recorded in many tropical and mild temperate regions of five distinct continents [29]. However, the climatic threshold that restricts medfly’s establishment is not certain yet. In Europe, the northernmost limit of the range expansion of this pest is considered to be Slovenia (above the 46th parallel north) [40]. However, continuous detections and infestations on late ripening hosts have been observed in Austria as well [41], and several recent detections have been recorded in Germany and other central European countries [42]. Furthermore, it has been estimated and predicted that in 2030 medfly will colonize the inland areas of France, southern Germany, Switzerland, Austria and Hungary, and it may be established in Northern France, central Germany, Poland and the Netherlands by 2050 [43]. In cooler temperate areas, the Mediterranean fruit fly overwinters mostly in the larval stage inside infested host fruits by prolonging its developmental duration from late autumn until spring. Hence, a limited portion of individuals withstands winter conditions and regenerates the entire population in spring and early summer [25]. Overwintering in human-made shelters in cooler areas of Northern Italy has been also demonstrated [44].

Low winter temperatures can harm insects since environmental temperature determines their body temperature. Both non-freezing and freezing injuries can be caused by exposure to low temperatures. The first type of injury is a corollary of short- or long-term exposure to low temperatures that induce transitions in the phase of the membrane lipids and disruption of ion balance [45]. During freezing injuries, many concurrent shifts happen, including dehydration and mechanical injury of cells [46,47,48]. The survival of several insect species during winter depends on mechanisms such as cold hardening to prepare for subfreezing temperatures [49]. Cold hardiness is the ability of an organism to survive or tolerate low temperatures and exposure periods (short or long) that are either suboptimal or lethal [46]. Furthermore, subfreezing temperatures could impose lethal damage due to the freezing of the organism’s body fluids [50,51]. Survival after short exposure to extreme thermal regimes is substantial for describing an insect’s lower lethal temperature [19,52,53]. The ability of insects to successfully overwinter depends not only on their response to short-term exposure to subfreezing temperatures (acute cold tolerance), but also to milder chilling for longer periods (chronic cold tolerance) [19,54]. Therefore, the study of insect thermal biology is essential to determine the ability of insects to invade and establish in new regions.

Thermal biology of the adults of some tephritids including *Bactrocera dorsalis* (Hendel) [1,55], *Bactrocera zonata* (Saunders) [56], *Bactrocera tryoni* (Froggatt) [57,58], *Bactrocera oleae* (Rossi) [59], *Bactrocera tau* (Walker) [60], *Ceratitis cosyra* (Walker) [61], *Ceratitis rosa* (Karsch) [62], *Ceratitis quilicii* (De Meyer, Mwatawala and Virgilio) [63], *Eurosta solidaginis* (Fitch) [64,65,66,67], *Rhagoletis cerasi* (Linnaeus) [68] and *Rhagoletis indifferens* (Curran) [69] has already been investigated. Regarding medfly, its broader distribution may justify its great cold tolerance, which might determine the establishment of populations and their invasion success [13,70,71]. Several studies have assessed the tolerance of adult medflies to extreme temperatures by examining different parameters of the insect’s thermal biology such as Critical Thermal Minimum (CTmin) and Critical Thermal Maximum CTmax [72,73,74,75], acute cold stress [13,71,76], chill coma recovery time (CCRT) [73,74,77,78,79], supercooling point (SCP) [13,80], heat knockdown time (HKDT) [73,74] and flight performance [81]. Thermal requirements and thresholds of the immature stages (eggs, larvae, pupae) of medfly have been sufficiently studied [82,83,84,85,86,87,88,89,90]. However, there is no comparative data regarding the response to extremely low temperatures of immatures originating from different populations, even though recent studies in medfly adults have demonstrated high variability in chill coma recovery time [77] and supercooling point (SCP) [13] among geographically divergent populations of the Northern Hemisphere.

The objective of this study was to estimate the cold tolerance of immature stages of different medfly populations of the Northern Hemisphere in an effort to gain insights in invasion dynamics and dispersal of this pest to northern, cooler areas. The results demonstrate extensive variability in the response of different populations depending on their developmental stage (i.e., egg, larva, pupa) and contribute to broadening the existing knowledge of the thermal biology of the Mediterranean fruit fly.

## 2. Materials and Methods

### 2.1. Populations

Three *C. capitata* populations originating from Greece (Volos, Crete) and Croatia (Dubrovnik) were tested (F_5_–F_13_ generations). To represent part of the climatic variation prevailing in different habitats of medfly in the temperate zone of the Northern Hemisphere we selected the three populations along a latitudinal gradient [91]. Analyses on bioclimatic variables have already proved the great variability in the climatic conditions among different regions, even if they pertain to the same climatic category [77].

### 2.2. Insect Rearing

The experiments were conducted in the laboratory of Entomology and Agricultural Zoology at the University of Thessaly, from May to December 2022. Small-scale rearing of medfly populations from Crete and Volos was established in the laboratory (25 ± 1 °C, 45–55% relative humidity, 14:10 Light:Dark photoperiod (photo phase beginning at 07:00 h)) from field-infested persimmons and bitter oranges, respectively. The population of Croatia was transported to our laboratory as pupae obtained from field-infested figs. Laboratory colonies of the different tested populations were established using initial numbers of 500–1000 pupae. Each colony was kept in 30 × 30 × 30 cm wood framed, wire-mesh cages under densities of approximately 100 adults per cage. Adults were provided with *ad libitum* access to water and food. The adult diet consisted of a mixture of yeast hydrolysate (YS), sugar and water (1:4:5 ratio) [92]. An artificial oviposition substrate (a pre-punctured, hollow plastic dome) was offered to females for egg laying [93]. The colonies were maintained by placing the domes in the rearing cages for 24 h and collecting the eggs with a soft paint brush from the inner surface of the dome. Approximately 100 eggs were horizontally deposited on the surface of cotton disks that had been previously submerged partially in the larval artificial diet and maintained in glass Petri dishes (9.5 cm Ø and 1.5 cm depth). The larval diet consisted of 50 g sugar, 50 g brewer’s yeast, 25 g soybean flour, 1 g salt mixture, 4 g ascorbic acid, 4 g citric acid, 0.75 g sodium propionate and 250 mL water) [94]. After the completion of larval development, the jumping larvae were pupated and kept in sterilized sand until adult emergence under identical laboratory conditions.

### 2.3. Assessment of LT_50_ at Subfreezing Temperatures

To determine the effect of low temperatures on the immature stages of the different *C. capitata* populations, preliminary experiments to estimate the lowest lethal temperature that kills 50% of individuals (LT_50_) were conducted. Different developmental stages were exposed for 1 h in a refrigerated, circulating programmable water-cooling bath (Polystat, Cole-Parmer containing silicone oil) with a ramping rate of 0.25 °C/min to a range of temperatures (0, −5, −8, −10, −12, −15, −18, −20 °C) for the eggs, (−2, −4, −5, −6, −8, −11 °C) for the larvae and (6, 4, 2, 0, −3, −5, −6, −8 °C) for the pupae (see details in Section 2.7). Using a Probit Analysis, the LT_50_ was estimated at −11 °C for the eggs, −4.4 °C for the larvae, and −5 °C for the pupae. Depending on availability of populations in the laboratory, flies from Crete were used in the preliminary trials for eggs, and flies from Volos were used in the preliminary trials for larvae and pupae, respectively. Additional details are given in the Supplementary Annexes (Tables S1, S2 and S3, respectively).

### 2.4. Effect of Subfreezing Temperatures on the Eggs

For our experiments, domes were placed for 24 h inside the colony cages of each population. Newly laid eggs were collected with a soft paint brush and placed on the surface of round black disks (5 cm Ø) made of soft lining fabric. Under the stereoscope, we placed 250 eggs of each population (5 disks/population, 50 eggs per disk). The eggs were placed in the center of the disk. Next, the disks were carefully folded diametrically opposed (such as a reticule looking form) and inserted in the middle of glass tubes (1.5 cm Ø and 15 cm depth). The top of each glass tube was covered with cotton wool and the tubes were submerged in the water bath (see details in Section 2.7). After the exposure, the eggs were placed in Petri dishes at 25 °C and the egg hatching rate was recorded for three consecutive days.

### 2.5. Effect of Subfreezing Temperatures on the L_3_ Instar Wandering Larvae

Regarding the larval stage, we collected the eggs following the procedure described in Section 2.2 and reared the larvae of the three different populations until the day they left the rearing medium to pupate. Wandering larvae, which abandoned the cotton disks and emerged from the sand to pupate, were promptly collected using soft sterilized forceps and placed directly in the bottom of the glass tubes, covering the gap at the top with cotton wool. Ten larvae were placed in each tube (replicate) and 10 replicates were considered for each population (100 larvae/population in total). After that, the glass tubes were submerged in the water bath (see details in Section 2.7). At the end of the exposure to low temperature, larvae were placed in Petri dishes filled with sterilized sand at 25 °C and the pupation rate was recorded for three consecutive days.

### 2.6. Effect of Subfreezing Temperatures on 4-Day-Old Pupae

Similarly, as in the two previous stages, we collected the eggs and allowed the larvae to complete their development; once they had pupated, we collected the pupae and placed them in Petri dishes. Four days after pupation, we randomly picked the pupae and recorded their weight using an analytical balance (resolution of 0.0001 g, Sartorius AG Göttingen, Germany BP 2105). We used 4-day-old pupae to ensure that the formation of the adult did not take place within the puparium [95,96]. Pupae with a weight less than 8 mg were rejected because the probability of being stressed or dead was increased [97,98,99]. Next, we transferred the pupae with soft forceps inside the glass tubes and finally we submerged them in the water bath (see details in Section 2.7). Ten pupae were placed in each tube (replicate) and 10 replicates were considered for each population (100 pupae/population in total). After the exposure, pupae were removed from the glass tubes, transferred to Petri dishes and kept at 25 °C, and the emergence rate of the adults was recorded for ten consecutive days.

### 2.7. Acute Cold Tolerance Assessment

Once we had calculated the LT_50_ of each developmental stage, we collected successively the aforementioned number of eggs, larvae and pupae of our 3 populations and placed them inside the glass tubes. We placed about 4 or 5 of the glass tubes in a floating tube rack and submerged them in the liquid (silicone oil) of the water bath for 1 h. To record the temperature inside the glass tubes, a type-T thermocouple sensor (36 SWG) was inserted into an empty tube. Thermocouples were connected to an 8-channel Picotech TC-08 (Pico Technology, Cambridge, UK) thermocouple interface that recorded accurately the temperature at 1 Hz via Picolog software (version 5.25.3) (www.picotech.com (accessed on 19 April 2022)). The method we used to assess cold tolerance of the different populations in each stage was identical with that described in Section 2.4, Section 2.5 and Section 2.6.

### 2.8. Data Analysis

All data analyses were performed using SPSS 26.0 (SPSS Inc., Chicago, IL, USA). The temperature–mortality data of the preliminary experiments were subjected to Probit analyses to estimate the LT_50_ of eggs, larvae and pupae, and evaluate the impact of 1 h exposure at low temperatures on these stages. The effects of subfreezing temperatures and control treatments on the mortality rates of immature stages of the different populations were analyzed with binary logistic regression analysis. For each developmental stage, analyses were conducted separately for subfreezing temperatures and separately for the control treatments. To determine the significance of the tested factors the Wald chi-square test (*χ*^2^) was used. In all tests the significance level was set at *α* = 0.05. Egg hatch rates and emergence rates were corrected using Abbott’s formula [100]. There was no need for correction on pupation rates since the control mortality in this case was <5% [101]. Graphs were generated in R version 4.2.1 [102] using the *ggplot2* (v3.3.6) [103] and *ggpubr* packages (v0.6.0) [104].

## 3. Results

### 3.1. Effect of Subfreezing Temperatures on the Eggs

**Figure 1:**
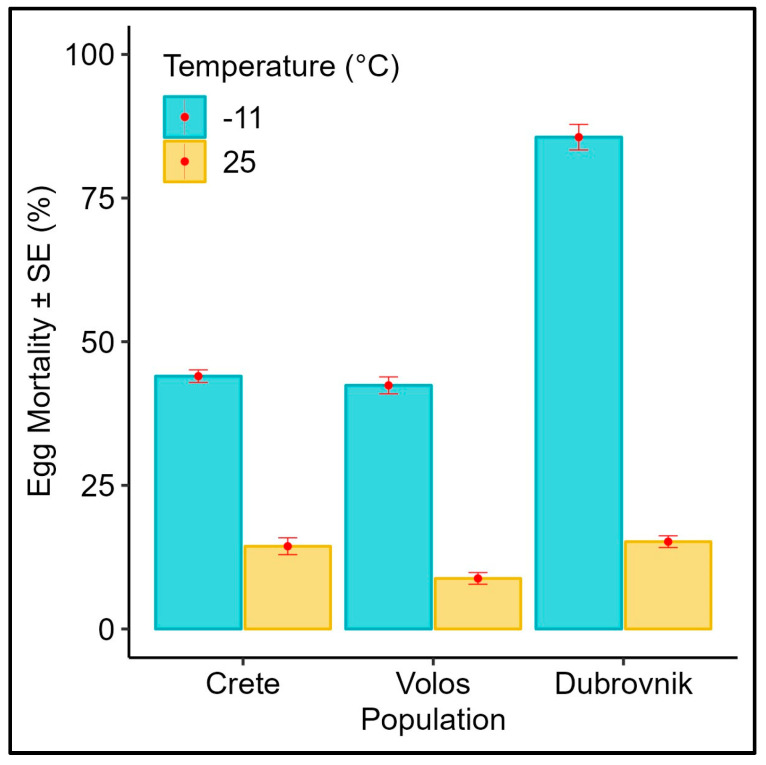
Average egg mortality (% ± SE) of different *Ceratitis capitata* populations exposed to −11 °C for 1 h (N = 250 eggs per treatment). The mortality of respective control eggs (kept at 25 °C) is also included.

The mortality rates of the eggs of different medfly populations exposed to a subfreezing temperature (−11 °C) and control (25 °C) are given in Figure 1. At −11 °C, population origin significantly affected larvae hatch rates (Table 1, *p* < 0.001), whereas all of the populations responded similarly at 25 °C (Table 1, *p* > 0.05). Greek populations did not exhibit any difference in acute cold stress and were more tolerant of low temperatures than the population from Croatia, which expressed a high mortality rate in the egg stage. Abbott’s corrected mortality for the egg stage is given in the Supplementary Annexes (Figure S1) as well.

### 3.2. Effect of Subfreezing Temperatures on the L_3_ Instar Wandering Larvae

**Figure 2:**
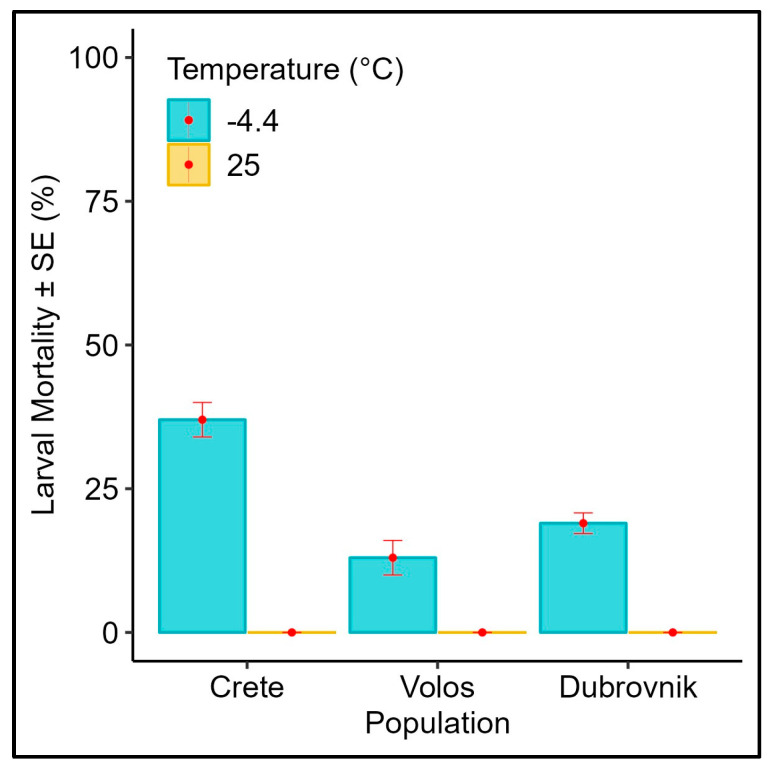
Average larval mortality (% ± SE) of different *Ceratitis capitata* populations exposed to −4.4 °C for 1 h (N = 100 larvae per treatment). The mortality of respective control larvae (kept at 25 °C) is also included.

Mortality rates of the larvae of different medfly populations exposed to a subfreezing temperature (−4.4 °C) and control (25 °C) are given in Figure 2. At −4.4 °C, population origin significantly affected pupation rates (Table 1, *p* < 0.001), whereas all larvae managed to pupate at 25 °C (no analysis was conducted since all larvae managed to pupate, indicating no significant differences among populations). The Greek population from Crete (the southernmost population we examined) was the only one expressing increased mortality at the larval stage while population from Volos was the least susceptible to the subfreezing temperature.

### 3.3. Effect of Subfreezing Temperatures on 4 Days Old Pupae

**Figure 3:**
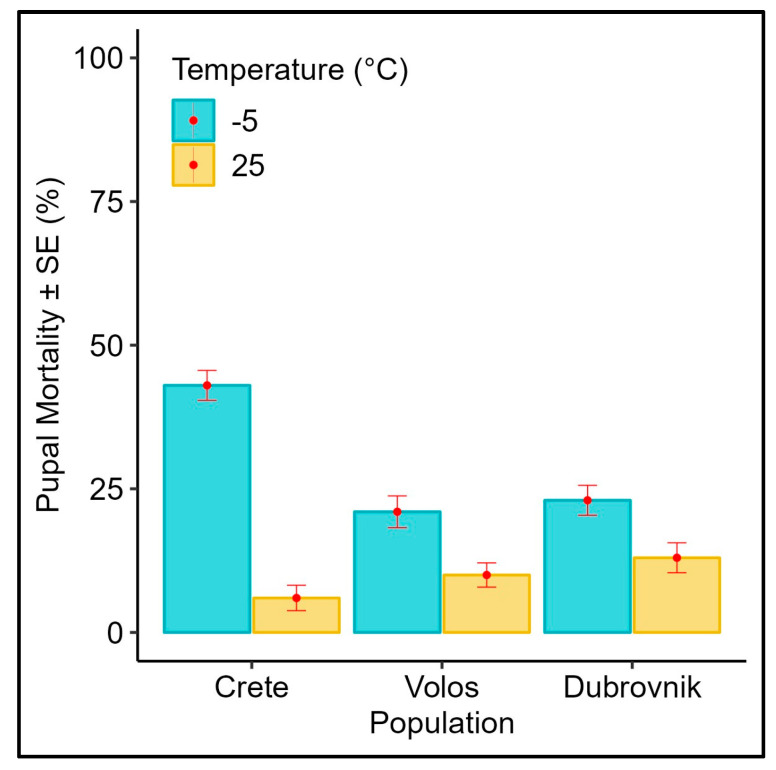
Average pupal mortality (% ± SE) of different *Ceratitis capitata* populations exposed to −5 °C for 1 h (N = 100 pupae per treatment). The mortality of respective control pupae (kept at 25 °C) is also included.

Mortality rates of the pupae of different medfly populations exposed to a subfreezing temperature (−5 °C) and control (25 °C) are given in Figure 3. At −5 °C, population origin significantly affected adult emergence rates (Table 1, *p* = 0.001), whereas all of the populations responded similarly at 25 °C (Table 1, *p* > 0.05). Pupae from Volos and Dubrovnik were the most cold-tolerant while pupae from Crete exhibited much higher mortality rates than the other two populations. Abbott’s corrected mortality for the pupal stage is given in the Supplementary Annexes (Figure S2) as well.

## 4. Discussion

We examined the effect of population origin and developmental stage on cold tolerance of the immature stages of three different medfly populations originating from the temperate region of Europe and demonstrated that (a) there are differences among populations in cold tolerance of all three developmental stages following short exposure of immatures to subfreezing temperatures, (b) each immature stage (egg, larva, pupa) responds differently to subfreezing temperatures and the egg stage seems to be the most tolerant at low temperatures.

Geographically divergent populations of medfly respond differently to subfreezing temperatures depending on their developmental stage. Indeed, there is a strong correlation among traits of thermal tolerance or resistance with the geographic distribution of different insect species [105]. Our results show that the egg is the most tolerant stage at low temperatures (LT_50_ = −11 °C), followed by pupae (LT_50_ = −5 °C) and then wandering larvae (LT_50_ = −4.4 °C). Likewise, experiments on the effect of low temperatures on different developmental stages of a closely related tephritid species (*B. dorsalis*) reveal the egg as the most cold-tolerant developmental stage [55]. The higher tolerance of eggs to extremely low temperatures may be attributed to the eggshell structure and the egg content that allow better protection against unfavorable conditions.

During oogenesis, sizeable numbers of proteins and lipids are incorporated into eggs [106]. Moreover, during vitellogenesis, the oocytes accumulate a significant number of lipid droplets along with triglycerides, phospholipids, granules of glycogen and some proteases [107]. The final, most proteinaceous, product of the follicle cells that surround the oocyte is the eggshell [108]. It is composed of the vitelline membrane, the wax layer, the innermost chorionic layer, the endochorion and the exochorion [109]. The endochorion is secreted by the follicle cells and contains proteins rich in proline [109] which has been established to have antifreeze protein activity (AFP) if suitable conditions are met [110,111]. Together, accompanied by the tuft, the compact endochorion and the granules all protect the micropylar canal from environmental shifts such as temperature and water entry [109,112]. This is important because the dense vitelline membrane in medfly gives greater protection to the micropylar apparatus where the head of the developing embryo is positioned [113]. In *Drosophila melanogaster* (Diptera: Drosophilidae), the wax layer is produced by the accumulation of vesicles filled with lipids between the follicle cells and the vitelline membrane. As these vesicles accumulate on the surface of membrane, they acquire a flat texture. Next, the vesicles are condensed into 3–4 levels of overlapping plaques building a watertight layer between the vitelline membrane and the chorion [114]. If the same structure exists in the wax layer of medfly, then it could probably play an important role in the level of susceptibility or tolerance of the eggs at low temperatures. Even though we detected differences in the cold tolerance of different populations at the egg stage, more research needs to be carried out οn the response of this stage to low temperatures in order to reveal potential physiological mechanisms that justify the high tolerance of medfly’s eggs.

Regarding larvae and pupae, the general metabolic rate during the metamorphosis of medfly follows a U-shaped curve [115,116], which reflects that the energy consumption peaks at the first stages of metamorphosis, decreases during the mid-pupal stage and then peaks again during the last hours of the pharate-adult stage. Lipids (mainly triacylglycerols) and carbohydrates (mainly glycogen and trehalose) comprise the basic sources of energy during metamorphosis [115]. During larval feeding activity, the reserves of accumulated glycogen are high and abruptly depleted immediately after the abandonment of the diet substrate and the prepupal stage. Specifically, the level of glycogen reaches its nadir during larval–pupal apolysis. The same happens with the levels of hemolymph carbohydrates (mainly trehalose). The nearly entire exploitation of glycogen and carbohydrates in these phases could be linked to the high energy demands during the wandering larva stage and the early pupa stage [117].

In our case, the increased susceptibility of the larvae may be a result of the high energy consumption and the cold protection molecules which are depleted during the wandering larva stage. Older studies have demonstrated the 2nd instar feeding larva as the most cold-tolerant stage (and then the 1st instar) facilitating the overwintering of medfly in cooler temperate areas inside infested fruit hosts, e.g., [118,119]. The lower metabolic rate and reduced activity accompanied by higher sugar content which are induced by subfreezing temperatures may account for the increased cold tolerance of 1st and 2nd instar larvae. Although the wandering larvae may not be the best candidates to test winter survival of medfly (since larvae leave the fruit to pupate and are directly exposed to low temperatures for a short period of time before pupal formation) [118] they can still provide interesting comparative results regarding thermal performance of different medfly populations. Testing 1st and 2nd instar larvae was technically and methodologically impossible because of their size and always being attached to larvae rearing medium. Simultaneously, the higher tolerance of the pupal stage compared to the larval one may be related to the pupa developmental stage that we considered in our experiments. The “mid-stage” pupae, according to the general metabolic rate during metamorphosis, do not require high levels of energy consumption, which may explain their higher tolerance due to the increased level of cryoprotectant reserves. This is in accordance with the findings of Papadogiorgou and colleagues [13], where the 1-day-old pupae expressed higher SCP than the 5-day-old ones. Energy reserves are crucial for the survival of extreme thermal conditions, as has been demonstrated for the wasp *Aphidius colemani* (Hymenoptera: Braconidae) where exposure to cold temperatures requires huge energy consumption by the insect to withstand these conditions, which would be difficult under depleted energy resources [120]. Furthermore, *Glossina pallidipes* (Diptera: Glossinidae) adults with the lowest chill coma values had the highest content in body lipids and water [121], indicating a connection between energy reserves and tolerance at low temperatures. Moreover, in *D. melanogaster* adults, high levels of cholesterol in the diet increased the quantity of cholesterol in the cell membranes and consequently the survival significantly increased after a 2 h cold shock at −5 °C, probably because of enhanced membrane fluidity [122]. Insect lipids are the elementary targets during physiological damage due to freezing and chilling. While low temperatures cause rigidification of phospholipid bilayers, membrane malfunction is very likely to happen. Due to the lipid rigidification there is also a decrease in the accessibility of fat stores for metabolic enzymes and consequently in their availability for basal metabolism [123].

Pupae were more cold-tolerant than larvae in our study which is in accordance with the study of Nyamukondiwa and colleagues [80]. Differences in LT_50_ estimates between pupae (the second most tolerant stage after eggs in our study) and that of Nyamukondiwa and colleagues [80] may be attributed to the different developmental stages of the pupae used for the experiments and other experimental details. In our case, the pupae were at the yellowish eyes stage (4 days old), which means that they were exposed to low temperatures at an earlier pupal developmental stage. Furthermore, experiments on the SCP of different developmental stages of seven medfly populations revealed that the pupal stage had the lowest SCP [13]. The different tolerance between pupae and larvae may be related to the potential involvement of the content of the alimentary channel (food, symbiotic bacteria, etc.) in ice formation [124], since the total deficiency of nucleating agents in the gut, hemolymph or other fluid compartments could explain the higher tolerance of pupae as well [125]. Hemolymph osmolality could be another determining parameter in the modulation of cryoprotectants (e.g., polyols and low molecular weight carbohydrates) produced by insects to mitigate harmful effects of low temperatures on cell organelle and membrane injuries to survive winter temperatures [126,127,128,129]. Some of the main roles of cryoprotectants are the hindrance of intracellular volume decrease below a critical minimum, the maintenance of the membrane bilayer structure and the decrease in SCP [48,127,130,131,132,133]. The accumulation of cryoprotectants may also be utilized by medfly during overwintering to increase the potential of its survival during winter [134].

Differences among the three populations were observed in all developmental stages. Larvae and pupae of the Cretan population were the most susceptible, which could be explained by their warmer southern origin. Interestingly, at the egg stage, the population of Dubrovnik was the least cold-tolerant, which is completely opposed to the above explanation on larvae and pupae. This could be explained by the fact that the microclimatic conditions [77,135] across each of these different areas can exert a strong impact on the cold tolerance of each population despite the latitude of its origin. Furthermore, this can be supported by the results of other assays comparing chill coma recovery [77] and SCP [13] of different medfly populations which originated from even more divergent locations than the populations in our study. These results demonstrated that despite the differences at each population’s latitude, there was no conclusive correlation between cold tolerance and climatic variability, suggesting the necessity of more investigation on this phenomenon. Furthermore, as far as any difference among populations is concerned, it might also be a consequence of different levels of maternal investment to the developing oocyte in the ovary during oogenesis depending on the population and its geographic distribution. Specifically, maternal genes operating in the nurse cells of the mother may pass different levels of maternal gene transcripts (proteins, mRNAs, tRNAs and organelles) to progeny [106,136]. However, this should be further investigated to elucidate this query with clarity.

## 5. Conclusions

When estimating cold tolerance of medfly, not only the developmental stage but also the population origin constitute significant parameters to consider in this pest. Cold tolerance of the different populations of geographically divergent regions around the globe is affected by both factors in various ways. According to our results, the egg stage seems to be the most cold-tolerant stage of medfly, followed by pupae and lastly by wandering larvae. Moreover, regarding larvae and pupae, their cold tolerance follows the pattern of their geographic location (i.e., the more southern, the more susceptible). This does not happen with the eggs where the most northern of our populations tend to be the least tolerant. Even though our three populations belong to the same climate category according to the Köppen-Geiger climate classification system, such differences among populations indicate potential microclimate differentiations across each of these distinct areas [77,135] and more research must be carried out to elucidate this query. Moreover, any similarities or differences among this and other studies may be a corollary of different methods and protocols followed in each case. For example, there are several studies dealing with the effect of low temperature on the tolerance of immature stages of medfly, but they deal mostly with disinfestation treatments via cold storage where much longer exposure times and higher temperatures are used than in our case [82,119,137,138,139,140]. In general, our findings are about to broaden the existing knowledge on cold tolerance of medfly and can further aid in better understanding plasticity and adaptive responses of this notorious pest, which is currently expanding its distribution limits to the north, increasingly threatening more fruit-producing areas of Europe and other regions of the Northern Hemisphere.

## Figures and Tables

**Table 1:** Summary results of binary logistic regression analysis on the effect of population origin on cold tolerance of the different developmental stages of medfly and their respective control treatments. *p* values higher than 0.05 are designated with ns (*ns* = non-significant).

Source of Variance	Dependent Variable	*χ*2	df	*p* Value
(−11 °C)				
Population	Egg Mortality	104.24	2	<0.001
(25 °C)				
Population	Egg Mortality	5.33	2	*ns*
(−4.4 °C)				
Population	Larval Mortality	16.62	2	<0.001
(25 °C)				
Population	Larval Mortality	*	*	*
(−5 °C)				
Population	Pupal Mortality	13.92	2	=0.001
(25 °C)				
Population	Pupal Mortality	2.72	2	*ns*

* No analysis was conducted since all larvae managed to pupate, indicating no significant differences among populations.

## Data Availability

The data presented in this study are available on request from the corresponding author. The data are not publicly available due to being part of a PhD thesis that is currently in progress.

## Supplementary Materials

The following supporting information can be downloaded at: https://www.mdpi.com/article/10.3390/biology12111379/s1, Table S1: LT_50_ value for the egg stage following Probit Analysis. Table S2: LT_50_ value for the larval stage following Probit Analysis. Table S3: LT_50_ value for the pupal stage following Probit Analysis. Figure S1: Egg mortality (%) of different *Ceratitis capitata* populations exposed to −11 °C for 1 hour (N = 250 eggs per treatment). The mortality of respective control eggs (kept at 25 °C) is also included. The red solid line gives the Abbott’s Corrected Mortality (%). Figure S2: Pupal mortality (%) of different *Ceratitis capitata* populations exposed to −5 °C for 1 hour (N = 100 pupae per treatment). The mortality of respective control pupae (kept at 25 °C) is also included. The red solid line gives the Abbott’s Corrected Mortality (%).
