# Supplementary material for: Differential Cold Tolerance on Immature Stages of Geographically Divergent Ceratitis capitata Populations
Source: Biology (Basel). 2023 Oct 27;12(11):1379. doi: 10.3390/biology12111379 (PMC10668952; doi:10.3390/biology12111379)
Supplement: Supplementary file 1 [file biology-12-01379-s001.zip › biology-2570045-supplementary.pdf]

Supplementary Materials

LT_{50} of the egg stage

We conducted preliminary experiments on the egg stage to estimate the lowest lethal temperature that kills 50% of the individuals (LT_{50}). We tested 8 different temperatures (0, -5, -8, -10, -12, -15, -18, -20°C). Specifically, newly laid eggs (24 hours) were placed on round black disks (5 cm Ø) made by soft lining fabric. There were 50 eggs per disk and 5 disks per population (overall 250 eggs per population). We used for this assessment a wildish population from Crete. Eggs were exposed for 1 hour at the different temperatures, in a refrigerated, circulating programmable water-cooling bath (Polystat, Cole-Parmer containing silicone oil) with ramping rate 0.25^{o}C/min. Following exposure, eggs were kept at 25°C and egg hatch rates were recorded for three consecutive days.

The LT_{50} value for the egg stage was estimated at -11°C following a Probit Analysis is (Table S1).

Confidence Limits

		95% Confidence Limits for Treatment		
	Probability	Estimate	Lower Bound	Upper Bound
PROBIT[a]	0,010	1,096	-3,742	16,757
	0,020	-0,328	-4,721	13,616
	0,030	-1,231	-5,351	11,632
	0,040	-1,910	-5,831	10,146
	0,050	-2,462	-6,226	8,941
	0,060	-2,933	-6,565	7,919
	0,070	-3,345	-6,866	7,026
	0,080	-3,714	-7,139	6,230
	0,090	-4,050	-7,389	5,508
	0,100	-4,359	-7,622	4,846
	0,150	-5,639	-8,617	2,137
	0,200	-6,656	-9,458	0,034
	0,250	-7,529	-10,230	-1,720
	0,300	-8,312	-10,980	-3,239
	0,350	-9,038	-11,737	-4,584
	0,400	-9,728	-12,526	-5,789
	0,450	-10,394	-13,368	-6,877
	0,500	-11,050	-14,281	-7,863
	0,550	-11,706	-15,280	-8,763
	0,600	-12,373	-16,380	-9,593
	0,650	-13,062	-17,596	-10,372
	0,700	-13,788	-18,950	-11,120
	0,750	-14,572	-20,476	-11,862
	0,800	-15,444	-22,236	-12,628
	0,850	-16,461	-24,345	-13,463
	0,900	-17,741	-27,058	-14,455
	0,910	-18,050	-27,721	-14,687
	0,920	-18,386	-28,443	-14,936
	0,930	-18,755	-29,241	-15,208
	0,940	-19,168	-30,134	-15,508
	0,950	-19,638	-31,157	-15,847
	0,960	-20,191	-32,362	-16,241
	0,970	-20,870	-33,850	-16,720
	0,980	-21,773	-35,834	-17,349
	0,990	-23,196	-38,976	-18,327

a. A heterogeneity factor is used.

Table S1. LT_{50} value for the egg stage following Probit Analysis.

LT_{50} of the larval stage

We also conducted preliminary experiments on the larval stage to estimate the lowest lethal temperature that kills 50% of the individuals (LT_{50}). We tested 6 different temperatures (-2, -4, -5, -6, -8, -11°C). Specifically, wandering larvae, which abandoned the cotton-disks and popped out in the sand to pupate were placed in the bottom of glass tubes covered with cotton wool. There were 10 larvae per tube and 10 tubes per population (overall 100 larvae per population). We used for this assessment a wildish population from Volos. Larvae were exposed for 1 hour at the different temperatures, in a refrigerated, circulating programmable water-cooling bath (Polystat, Cole-Parmer containing silicone

oil) with ramping rate 0.25°C/min. After the exposure, larvae were kept at 25°C and the pupation rates were recorded for three consecutive days.

The LT_{50} value for the larval stage was estimated at -4.4°C (Table S2).

Confidence Limits

	Probability	95% Confidence Limits for Treatment		
		Estimate	Lower Bound	Upper Bound
PROBIT[a]	0,010	-3,144	-3,837	2,287
	0,020	-3,293	-3,932	1,560
	0,030	-3,387	-3,993	1,100
	0,040	-3,458	-4,040	0,755
	0,050	-3,516	-4,079	0,475
	0,060	-3,565	-4,113	0,237
	0,070	-3,608	-4,143	0,029
	0,080	-3,647	-4,171	-0,156
	0,090	-3,682	-4,196	-0,325
	0,100	-3,714	-4,220	-0,479
	0,150	-3,848	-4,325	-1,113
	0,200	-3,954	-4,416	-1,609
	0,250	-4,045	-4,504	-2,025
	0,300	-4,127	-4,594	-2,388
	0,350	-4,203	-4,689	-2,712
	0,400	-4,275	-4,795	-3,003
	0,450	-4,345	-4,917	-3,266
	0,500	-4,413	-5,061	-3,501
	0,550	-4,482	-5,233	-3,708
	0,600	-4,552	-5,438	-3,888
	0,650	-4,624	-5,680	-4,043
	0,700	-4,699	-5,965	-4,178
	0,750	-4,781	-6,297	-4,298
	0,800	-4,873	-6,689	-4,409
	0,850	-4,979	-7,166	-4,519
	0,900	-5,113	-7,786	-4,638
	0,910	-5,145	-7,938	-4,665
	0,920	-5,180	-8,104	-4,693
	0,930	-5,219	-8,287	-4,723
	0,940	-5,262	-8,492	-4,756
	0,950	-5,311	-8,728	-4,792
	0,960	-5,369	-9,005	-4,833
	0,970	-5,440	-9,348	-4,883
	0,980	-5,534	-9,805	-4,947
	0,990	-5,683	-10,529	-5,044

a. A heterogeneity factor is used.

Table S2. LT_{50} value for the larval stage following Probit Analysis.

LT_{50} estimation of the pupal stage

Preliminary experiments on the pupal stage to estimate the lowest lethal temperature that kills 50% of the individuals (LT_{50}) were conducted as well. We tested 8 different temperatures (6, 4, 2, 0, -3, -5, -6, -8°C). Pupae of 4 days old were randomly picked, weighed, and placed in the bottom of glass tubes covered with cotton wool. There were 10 pupae per tube and 10 tubes per population (overall 100 pupae per population). We used for this assessment wildish population from Volos. Pupae were exposed at the different temperatures for 1 hour in a refrigerated, circulating programmable water-cooling bath (Polystat, Cole-Parmer containing silicone oil) with ramping rate 0.25°C/min. Following exposure, pupae were kept at 25°C and the pupation rate was recorded for ten consecutive days.

The LT_{50} value for the pupal stage was estimated at -5°C (Table S3)

Confidence Limits

	Probability	95% Confidence Limits for Treatment		
		Estimate	Lower Bound	Upper Bound
PROBIT[a]	0,010	9,175	3,619	44,021
	0,020	7,516	2,578	37,584
	0,030	6,463	1,902	33,517
	0,040	5,671	1,382	30,468
	0,050	5,027	0,949	27,998
	0,060	4,478	0,572	25,904
	0,070	3,998	0,234	24,075
	0,080	3,567	-0,075	22,446
	0,090	3,176	-0,364	20,970
	0,100	2,815	-0,637	19,619
	0,150	1,323	-1,870	14,129
	0,200	0,137	-3,044	9,960
	0,250	-0,880	-4,309	6,641
	0,300	-1,794	-5,782	3,997
	0,350	-2,640	-7,543	1,943
	0,400	-3,444	-9,599	0,380
	0,450	-4,221	-11,900	-0,822
	0,500	-4,986	-14,386	-1,782
	0,550	-5,751	-17,022	-2,593
	0,600	-6,528	-19,803	-3,315
	0,650	-7,331	-22,749	-3,988
	0,700	-8,178	-25,909	-4,644
	0,750	-9,091	-29,361	-5,308
	0,800	-10,109	-33,240	-6,013
	0,850	-11,294	-37,795	-6,801
	0,900	-12,787	-43,560	-7,759
	0,910	-13,147	-44,957	-7,987
	0,920	-13,538	-46,475	-8,232
	0,930	-13,969	-48,147	-8,500
	0,940	-14,450	-50,015	-8,798
	0,950	-14,998	-52,148	-9,136
	0,960	-15,642	-54,657	-9,530
	0,970	-16,434	-57,744	-10,012
	0,980	-17,487	-61,852	-10,648
	0,990	-19,146	-68,335	-11,642

a. A heterogeneity factor is used.

Table S3. LT_{50} value for the pupal stage following Probit Analysis.

Graphs including Abbott's Corrected Mortality for the egg and pupal stage (no correction on larval mortality was applied since the control mortality in this case was < 5%)

Figure S1

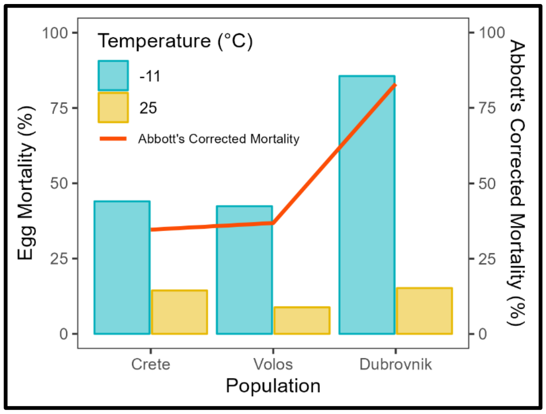

Figure S1. Egg mortality (%) of different *Ceratitis capitata* populations exposed to -11°C for 1 hour (N = 250 eggs per treatment). The mortality of respective control eggs (kept at 25°C) is also included. The red solid line gives the Abbott's Corrected Mortality (%).

Figure S2

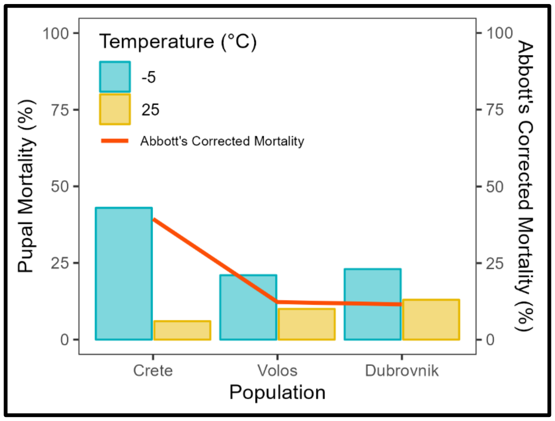

Figure S2. Pupal mortality (%) of different *Ceratitis capitata* populations exposed to -5ºC for 1 hour (N = 100 pupae per treatment). The mortality of respective control pupae (kept at 25ºC) is also included. The red solid line gives the Abbott's Corrected Mortality (%).
